# Qualitative Profiling, Antioxidant and Antimicrobial Activities of Polar and Nonpolar Basil Extracts

**DOI:** 10.3390/foods13182993

**Published:** 2024-09-21

**Authors:** Vera Vidaković, Bojan Vujić, Milka Jadranin, Irena Novaković, Snežana Trifunović, Vele Tešević, Boris Mandić

**Affiliations:** 1 Department of Ecology, University of Belgrade—Institute for Biological Research “Siniša Stanković”—National Institute of the Republic of Serbia, Bulevar despota Stefana 142, 11108 Belgrade, Serbia; vera.vidakovic@ibiss.bg.ac.rs; 2 University of Belgrade—Faculty of Chemistry, Studentski trg 12–16, 11000 Belgrade, Serbia; bvujic@gmail.com (B.V.); snezanat@chem.bg.ac.rs (S.T.); vtesevic@chem.bg.ac.rs (V.T.); 3 University of Belgrade—Institute of Chemistry, Technology and Metallurgy, Department of Chemistry, Njegoševa 12, 11000 Belgrade, Serbia; milka.jadranin@ihtm.bg.ac.rs (M.J.); irena.novakovic@ihtm.bg.ac.rs (I.N.)

**Keywords:** sweet basil, antioxidant potential, antimicrobial activity, flavonoid, phenolic acid, terpenoid, fatty acid, solvent polarity, HPLC-MS

## Abstract

Basil (*Ocimum basilicum* L.) is a widely used culinary herb. In this study, ethanol, dichloromethane, and sunflower oil were used separately as solvents with distinct polarities for the extraction of basil aerial parts to simulate the different polarity conditions in domestic food processing. The oil extract (OE) was re-extracted with acetonitrile, and the chemical composition, antioxidant potential, and antimicrobial activities of the ethanol (EE), dichloromethane (DCME), and acetonitrile (ACNE) extracts were determined. A total of 109 compounds were tentatively identified in EE, DCME, and ACNE by HPLC–DAD/ESI-ToF-MS. Fatty acids were present in all extracts. Phenolic acids and flavonoids dominated in EE. DCME was characterised by triterpenoid acids, while diterpenoids were mainly found in ACNE. The extracts were analysed for their antioxidant capacity using the 2,2-diphenyl-1-picrylhydrazyl radical (DPPH) assay. EE and DCME showed significant radical scavenging potential. Antimicrobial activity was explored in eight bacterial, two yeast, and one fungal species. All extracts exhibited high antifungal activity, comparable to or better than that of the commercial drug nistatin. Antibacterial activities were notable for EE and ACNE, while DCME showed no activity against bacteria in the applied concentration ranges. The different polarities of the solvents led to distinctive phytochemical compositions and bioactivities in the extracts.

## 1. Introduction

A healthy diet through the consumption of certain foods and beverages is an important link in the promotion of health and the prevention and management of diseases. For example, significant positive results have been achieved in cardiovascular disease and type 2 diabetes by changing dietary habits, e.g., by incorporating fruits, vegetables, whole grains and low-fat dairy products [1]. In this regard, plants play an important role as a source of nutrients and specialised metabolites (formerly known as secondary metabolites). Specialised metabolites are classified into four main groups, namely terpenoids, phenolic compounds, alkaloids, and sulfur-containing compounds [2], and generally represent bioactive compounds that are associated with valuable health-enhancing properties, such as antioxidant, antimicrobial, anti-inflammatory, and anticancer properties [3,4,5,6,7,8], but can also have adverse effects, including neurotoxic, hepatotoxic, mutagenic, and carcinogenic effects [9,10,11,12].

*Ocimum basilicum* L., commonly known as basil or sweet basil, is a highly aromatic herb that is widely used to enhance the flavour and aroma of various culinary dishes. It belongs to the Lamiaceae family and is native to tropical regions from Central Africa to Southeast Asia. Although it is considered an annual plant, it can also be grown as a biennial or perennial in regions with tropical or Mediterranean climates [13].

Basil leaves are commonly used as a fresh or dried popular spice. Apart from its culinary use, basil has a long history as a medicinal plant. Traditionally, it has been used to treat various ailments, such as headaches, coughs, gastrointestinal problems, menstrual irregularities, skin conditions, and kidney dysfunction [14,15]. The diverse bioactive compounds in basil contribute to its remarkable medicinal, pharmaceutical, and health-promoting properties. Basil essential oils, generally characterised by terpenoids and aromatic phenylpropanoid compounds, have insecticidal, nematicidal and antimicrobial properties and are used in the food, pharmaceutical, and cosmetic industries [16,17]. Essential oils from *Ocimum sanctum* and *O. basilicum*, as well as their main constituents eugenol and linalool, respectively, exhibit strong nematicidal activity against *Meloidogyne incognita* larvae [18]. Linalool and methylchavicol from the essential oil of *O. basilicum* displayed fungistatic activity against *Rhizopus nigricans*, while eugenol and cineole had a strong inhibitory effect on *Fusarium oxysporum* f. sp. *vasinfectum* [19]. In addition to essential oils, basil also contains a number of non-volatile constituents, including fatty acids, flavonoids, phenols, terpenoids, and steroids [20]. These non-volatile constituents contribute to the therapeutic efficacy of basil, including its immunomodulatory, antioxidant, anxiolytic, adaptogenic, and antidiabetic properties [17,20]. Quercetin-3-*O*-glucoside, a flavonoid commonly found in *Ocimum* species, showed antibacterial and antioxidant activities [21]. Other flavonoids, such as rutin, luteolin-7-*O*-glucoside, orientin and kaempferol, have been shown to possess antiulcer, anti-inflammatory, cytotoxic and gastroprotective activities, while the triterpenoid acids ursolic acid, 3-*epi*-maslinic acid and oleanolic acid exhibit antihyperglycemic, hepatoprotective and leishmanicidal effects, respectively (reviewed in [21]).

The preparation and processing of food can have a major impact on the extraction and bioavailability of nutrients. Different polarity media lead to different extraction of bioactive compounds. In addition, bioavailability depends on the food matrix. For example, it has been shown that the fat-soluble vitamin A precursors are better absorbed from high-fat foods [22]. The aim of this study was to investigate the influence of solvents with different polarities, i.e., ethanol, dichloromethane, and sunflower oil, on the phytochemical composition, antioxidant potential, and antimicrobial activities of extracts to simulate different food preparation conditions. Ethanol was used to simulate more polar food preparation conditions, such as broth cooking. Oil simulated apolar conditions, such as those achieved during stir-frying, braising/baking with vegetable oil, or marinating in oil. Since the oil extracts could not be analysed directly with HPLC, they were re-extracted with acetonitrile, which is relatively polar and probably could not extract all oil extractives. Therefore, the extraction of basil with dichloromethane was performed to identify the less polar extractives.

## 2. Materials and Methods

### 2.1. Plant Material

A commercial sample of Ocimum basilicum was obtained from the Institute for Medicinal Plant Research “Dr. Josif Pančić”, Belgrade, Serbia, lot number 06640319. Aerial parts of wild-growing plants were collected during full bloom.

### 2.2. Extraction Procedure

The dried and powdered plant material (18 g) was extracted separately with 50 g of 96% ethanol, 100 g of dichloromethane and 100 g of sunflower oil (commercial product “Dijamant”, DOO Zrenjanin, Zrenjanin, Serbia) in a dark place at room temperature for seven days. The extracts obtained were filtered. The oil extract (33 mL) was re-extracted with 20 mL of acetonitrile using an overhead rotary mixer for 18 h at room temperature. The solvent was removed from the extracts by rotary evaporation. Prior to HPLC–DAD/ESI–ToF-MS analysis, the extracts were dissolved in methanol to a final concentration of 10 mg/mL and filtered through a 0.45 μm pore-size filter.

### 2.3. HPLC–DAD/ESI–ToF-MS Analysis

The chemical composition of the extracts was analysed by an HPLC apparatus (Agilent 1100 Series, Agilent Technologies, Waldbronn, Germany) with a degasser, autosampler, LiChrospher 100 RP18e column (250 × 4.0 mm i.d.; 5 μm) and a DAD detector in combination with a 6210 Time-of-Flight LC/MS System (Agilent Technologies, Santa Clara, CA, USA). The mobile phase consisted of 0.2% formic acid solution in water (solvent A) and acetonitrile (solvent B). The isocratic and gradient elution programme at a flow rate of 1.00 mL/min was as follows: 0–5 min 10–20% B, 5–10 min 20% B, 10–20 min 20–30% B, 20–30 min 30–70% B, 30–35 min 70–100% B, 35–40 min 100% B, 40–41 min 100–10% B, 41–45 min 10% B. The injection volume was 10 μL and the column was thermostated at 25 °C. Signals were detected in the 190–550 nm wavelength range by a DAD. The charged molecular ions were obtained by electrospray ionisation (ESI) in the negative ionisation mode at atmospheric pressure. The ionisation source conditions were as follows: capillary voltage, 4000 V; gas temperature, 350 °C; drying gas flow rate, 12 L/min; nebuliser pressure, 45 psig (310.26 Pa); fragmentation voltage, 140 V. Masses were measured in the range 100–2500 *m*/*z*. MassHunter Workstation software (version A.02.02, Agilent Tchnologies) was used for data recording and processing.

### 2.4. Antioxidant Assay

Free radical scavenging activity of plant extracts was evaluated by 2,2-diphenyl-1-picrylhydrazyl (DPPH) assay [23]. Polar ethanol extract (EE) was analysed by the polar DPPH method (DPPH in methanol) and nonpolar extracts, i.e., dichloromethane (DCME), acetonitrile (ACNE) and oil (OE) extracts were analysed by the nonpolar DPPH method (DPPH in toluene). The concentrations of the extracts ranged from 0.1 to 1.75 mg/mL. A mixture of an extract solution (200 µL) and a 0.1 mM solution of DPPH (1800 µL) was shaken and incubated in the dark for 30 min. The absorbance of the remaining DPPH radical was measured at 517 nm (A_sample_). All samples were prepared in triplicate. The percentage of inhibition of the DPPH radical, I(%), by each sample was calculated according to the following equation:

$$I\left(\%\right)=\frac{A_{blank}-A_{sample}}{A_{blank}}\times 100,$$

where A_blank_ is the absorbance of DPPH with methanol or toluene instead of the extract solution.

The EC_50_ value (concentration of the extract that reduces the absorption of the DPPH solution by 50%) was calculated using the curve of the dependence of I(%) on the concentration of each extract. Butylated hydroxytoluene (BHT), a known artificial antioxidant, was dissolved in methanol (for the polar method) or toluene (for the nonpolar method) and used as a positive probe.

To compare the antioxidant capacity of the extracts, the results were also expressed in BHT equivalents (BHTE), i.e., µg BHTE/mg extract, which was calculated as follows:

$$BHTE=\frac{EC50\;BHT(\frac{mg}{mL})}{EC50\;extract(\frac{mg}{mL})}\times 1000,$$

where the EC_50_ of BHT in methanol was used for the calculations for EE, and the EC_50_ of BHT in toluene was used for the calculations for DCME, ACNE, and OE.

### 2.5. Antimicrobial Assay

Antimicrobial activity was tested against Gram-negative bacteria *Escherichia coli* (ATCC 25922), *Pseudomonas aeruginosa* (ATCC 9027), *Proteus hauseri* (ATCC 13315), *Klebsiella pneumoniae* (ATCC 10031), *Salmonella enterica* subsp. *enterica* serovar Enteritidis (ATCC 13076), Gram-positive bacteria *Staphylococcus aureus* (ATCC 6538), *Bacillus subtilis* (ATCC 6633), *Clostridium sporogenes* (ATCC 19404), yeasts *Candida albicans* (ATCC 10231), *Saccharomyces cerevisiae* (ATCC 9763) and the fungal strain *Aspergillus brasiliensis* (ATCC 16404). These strains of microbes were selected as the most common causes of infections in humans.

Antimicrobial activity was evaluated using the broth microdilution method according to the National Committee for Clinical Laboratory Standards [24], as described by Vujić et al. [25]. The 96-well plates were prepared by adding 100 μL of Mueller-Hinton broth for bacteria and Sabouraud dextrose broth for yeasts and fungi to each well. The test extracts were dissolved in DMSO to a stock concentration of 20 mg/mL, then 100 μL of the stock solution of the tested extracts was added to the first row of the plate and diluted twice in the broth. The direct colony method was used to prepare the bacterial and yeast suspension in sterile 0.9% saline, while spores from agar slants with growing Aspergilli were carefully stripped into sterile 0.9% saline to prepare the fungal spore suspension. The turbidity of the suspension was determined by comparison with the 0.5 McFarland standard. After measuring the optical density OD_600_, the colony count was also checked after a series of dilutions of the initial suspensions. Due to the visual detection of growth inhibition, the maximum concentrations of the microorganisms were used. Ten µL of bacterial or yeast suspension or spore suspension was added to each well to achieve a final concentration of 10^6^ CFU/mL for bacteria and 10^5^ CFU/mL for yeasts and fungi. Chloramphenicol served as a positive control for bacteria, while nystatin served as a positive control for yeasts and fungi. The inoculated plates were incubated at 37 °C for 24 h for bacteria and at 28 °C for 48 h for yeasts and fungi. The minimum inhibitory concentration (MIC) was determined as the lowest concentration that inhibited visible microbial growth.

Minimum bactericidal (MBC) and minimum fungicidal concentrations (MFC) were determined by plating 10 μL of samples from wells where no colony growth was observed onto nutrient agar medium for bacteria and Sabouraud dextrose agar for yeasts and fungi. After the incubation period, the lowest concentration with no visible growth (no colony) was defined as the minimum microbicidal concentration.

### 2.6. Statistical Analysis

The antioxidant properties of the extracts were compared by analysis of variance (ANOVA) using the statistical software R, version 4.2.2 [26]. The significance level was set at *p* ≤ 0.05, and the type I error rate in hypothesis testing was controlled using the Bonferroni *p*-adjustment method.

## 3. Results

### 3.1. Phytochemical Profile

Three solvents of different polarities were used for the extraction of *O. basilicum* aerial parts. Ethanol, dichloromethane and acetonitrile oil extract gave yields of 2.70%, 3.07%, and 0.89% of dry weight, respectively. In total, 109 compounds were tentatively identified by HPLC–DAD/ESI-ToF-MS based on their exact molecular masses and corresponding molecular formulas, UV spectra (where available, depending on content and/or molar absorption coefficient), and literature data on *Ocimum* species and/or Lamiaceae family (Table 1).

**Figure 1:**
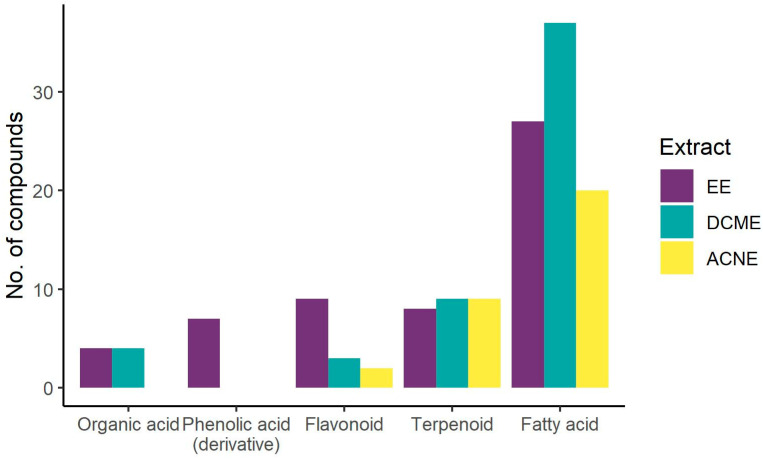
Distribution of representative compound classes tentatively identified in *O. basilicum*. EE—ethanol extract, DCME—dichloromethane extract, ACNE—acetonitrile oil extract.

**Figure 2:**
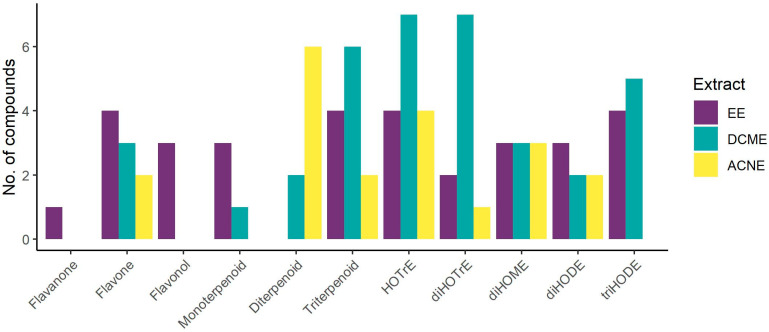
Distribution of representative subgroups of flavonoids, terpenoids, and fatty acids tentatively identified in *O. basilicum*. HOTrE—hydroxyoctadecatrienoic acid; diHOTrE—dihydroxyoctadecatrienoic acid; diHOME—dihydroxyoctadecenoic acid; diHODE—dihydroxyoctadecadienoic acid; triHODE—trihydroxyoctadecadienoic acid; EE—ethanol extract; DCME—dichloromethane extract; ACNE—acetonitrile oil extract.

Phytochemical analysis revealed the presence of organic acids, phenolic acids, flavonoids, terpenoids and fatty acids as the most represented compounds (Figure 1). EE was characterised by fatty acids, flavonoids, phenolic acids and monoterpenoids. Flavone and flavonol derivatives were the predominant flavonoids (Figure 2). Being the most hydrophilic, phenolic acids and glycosylated flavonoids were found only in EEs, while the organic acids were present in EEs and DCMEs. Terpenoids and fatty acids were the main compound classes identified in DCME and ACNE. Terpenoids were represented mostly by triterpene acids in DCME, diterpenoids in ACNE and monoterpenoid glucosides in EE (Table 1, Figure 2). The majority of the fatty acids identified in the extracts were (poly)unsaturated hydroxyoctadecatrienoic (HOTrE), dihydroxyoctadecatrienoic (diHOTrE), dihydroxyoctadecenoic (diHOME), dihydroxyoctadecadienoic (diHODE) and trihydroxyoctadecadienoic (triHODE) acids (Table 1, Figure 2).

### 3.2. Antioxidant Potential

Table 2 presents the results of the antioxidant activity of the basil extracts evaluated by the 2,2-diphenyl-1-picrylhydrazyl (DPPH) radical assay. The scavenging effects of the extracts are expressed as EC_50_ values. A lower EC_50_ value indicates that the sample displays higher antioxidant activity. EE exhibited five times weaker antioxidant potential than the BHT standard (methanol), while DCME exhibited seven times lower activity than BHT (toluene).

The antioxidant capacity of the extracts was compared on the basis of BHTE by ANOVA. A higher BHTE value means that the sample has a higher antioxidant activity. The extracts showed decreasing antioxidant capacity, as follows: EE > DCME > ACNE > OE (Table 3).

### 3.3. Antimicrobial Properties

The antimicrobial activities of *O. basilicum* extracts are listed in Table 4, Table 5 and Table 6. The extracts displayed notable antibacterial activity in the range of 0.313–2.5 mg/mL, except DCME, which showed no antibacterial activity against all the tested bacteria over the concentration range investigated. EE exhibited the strongest activity against a Gram-negative bacterium *Pseudomonas aeruginosa*, while ACNE was most potent against a Gram-positive *Staphylococcus aureus* and a Gram-negative *Proteus hauseri*, as indicated by the lowest minimum inhibitory concentration (MIC) values. All extracts displayed better or the same antifungal activity compared to the commercial drug nystatin.

## 4. Discussion

### 4.1. Phytochemical Profile

In this study, qualitative analyses of *O. basilicum* aerial parts extracted with solvents of different polarities were performed to gain insight into the chemical composition and bioactivity of food extracts prepared under different cooking conditions, taking into account that more polar conditions lead to the extraction of more polar components and, conversely, nonpolar conditions lead to the extraction of nonpolar components. To the best of the authors’ knowledge, this is the first comprehensive, untargeted analysis of extracts from basil aerial parts with gradient polarity; for the first time, 109 compounds belonging to ten different compound classes were tentatively identified from the same plant material. The results highlight the differences in the phytochemical composition between the ethanol, dichloromethane and sunflower oil basil extracts. Ethanol was the superior solvent for extracting phenolic acids and flavonoids, which is in accordance with previous findings. All phenolic acids tentatively identified in the present work, i.e., protocatechuic acid (compound **6**, Table 1), 4-hydroxybenzoic acid (**7**), caffeic acid (**8**), *p*-coumaric acid (**9**), rosmarinic acid (**10**), and salvianolic acids (**11**, **12**), have been previously reported in *O. basilicum* [13,14,48,85,86]. Rosmarinic acid is frequently found in medicinal plants of the Lamiaceae family [87]. It has been reported to be one of the predominant phenolic compounds in *O. basilicum* [14,85,88]. It has demonstrated a myriad of biological and pharmacological activities in prior research, including antioxidant, anti-inflammatory, antimutagenic, cytotoxic, neuroprotective, antimicrobial and immunomodulatory effects from in vitro studies; anti-inflammatory, antitumour, antithrombotic, antivenom, and protective effects from in vivo studies; and anti-inflammatory effects in treating several ailments from clinical trials, as reviewed by Amoah et al. [87]. It is believed that many of these effects are supported by the antioxidant and radical scavenging properties of rosmarinic acid.

Among the flavonoids, six glycosylated flavonoids (**13, 14, 20–23**) and five aglycones, including apigenin (**15**) and four methoxylated compounds (**16–19**), were found. Apigenin and glycosylated flavonoids were restricted to the ethanol extract, while the methoxylated derivatives were also extracted with dichloromethane and sunflower oil. Natural flavonoids are mostly found in *O*-glycoside or *C*-glycoside forms in plants. Glycosylation increases the chemical stability and solubility of flavonoids and provides access to active membrane transport systems that recognise glycosylated compounds but not their aglycones [89]. Methoxylated flavonoids are lipophilic molecules commonly found on the plant surface, where they are assumed to provide protection against harmful UV radiation and microbial infection, and there is evidence for their herbivore deterrence role [39,89]. Although methylation reduces the hydrophilicity and antioxidant potential of flavonoids in vitro, it increases their bioavailability by blocking the hydroxyl functional groups involved in the further catabolism of these compounds in living organisms. *O*-Methylated compounds generally exhibit higher in vivo anticancer activity than their corresponding hydroxylated derivatives [90]. In addition, methoxylated flavonoids can permeate membranes more easily due to their increased hydrophobicity, which facilitates their interaction with microorganisms in antimicrobial defence.

Ethanol and dichloromethane were the most effective solvents for the isolation of triterpenoids (**37–45**), among which the majority were triterpenoid acids, which have proven hepatoprotective, anti-inflammatory, antirheumatic, antiviral, antioxidant, and antitumour activities [76,91,92]. On the other hand, sunflower oil was superior in the isolation of diterpenoid compounds (**28–35**). Labdane, abietane and *ent*-kaurene diterpenoids, with prominent biological activities such as antioxidative, gastroprotective, and cytotoxic activities [70,93,94], were tentatively identified in the present study.

In all three extracts that were examined, a variety of fatty acids were found, particularly unsaturated hydroxy fatty acids. These types of fatty acids are a significant component of plant seed oils and waxes. The presence of polyunsaturated fatty acids, which are considered important health promoters due to their anti-inflammatory, antioxidant, and cytotoxic properties, aligns with previous studies on the fatty acid composition of basil leaves [95,96].

### 4.2. Antioxidant Potential

Different compositions of phytochemicals led to variations in the antioxidant effects of the extracts. The highest antioxidant activities were obtained with ethanol extracts, which is consistent with previous studies showing the superiority of polar protic solvents in obtaining extracts of *O. basilicum* with high antioxidant potential [86,88,97]. The ability of polar extracts to scavenge radicals is attributed to their phenolic content, particularly that of the phenolic acids and flavonoids. Their mode of action may include the inhibition of enzymes/chelation of trace elements involved in the formation of reactive oxygen species or the reduction of highly oxidising free radicals by hydrogen atom donation [25]. The phenoxyl radical formed by hydrogen atom donation is stabilised by delocalisation via a conjugated aromatic ring system. Particularly pronounced antioxidant activities are observed in phenolic acids possessing two hydroxyl groups at the *ortho* position [16], as found in protocatechuic (**6**), caffeic (**8**), rosmarinic (**10**), and salvianolic acids (**11, 12**) in the present study, as well as in certain flavonoids with catechol groups. The second oxygen atom of the hydroxyl group in the *ortho* position is also involved in delocalisation, which further stabilises the phenoxyl radical [98]. Furthermore, the catechol group can scavenge two radicals and chelate transition metal ions, which are involved in the formation of free radicals [99]. In addition to flavonoids and phenolic acids, some diterpene compounds, such as carnosic acid, a phenolic abietane diterpene found in ACNE, and other abietane diterpenes found in DCME and ACNE (**28, 30, 31, 34, 35**) have also been reported to exhibit remarkable antioxidant activities [93,94].

### 4.3. Antimicrobial Properties

A large number of infections of the urinary tract, gastrointestinal tract and respiratory tract are caused by bacteria [100,101,102]. Some of the microbes used in this work are causative agents of a variety of diseases and are associated with the problem of the emergence of resistant strains [103]. These microbial strains were selected as the most common causes of infections in humans. For example, *Escherichia coli*, *Klebsiella pneumoniae*, *Staphylococcus aureus*, *Pseudomonas aeruginosa*, *Proteus* spp. and *Candida* spp. are among the most common causes of urinary tract infections [104], while *Salmonella* spp., *Clostridium* spp., *E. coli*, *Candida* spp. and *Aspergillus* spp. are microbial pathogens that cause more severe or complicated gastrointestinal infections in immunocompromised hosts [101].

There are numerous studies on the antimicrobial effect of *O. basilicum* extracts, most of which investigated the effect of the essential oils and the ethanol, methanol or water extracts of the leaves. Backiam et al. [105] found that ethanol and methanol extracts of basil leaves inhibited the growth of the bacterial species *K. pneumoniae*, *P. aeruginosa*, *E. coli* and *S. aureus* investigated herein. Against *K. pneumoniae* they were as effective as the control antibiotic ampicilin; against *P. aeruginosa* the ethanol extract was better than the control and against *E. coli* and *S. aureus*, slightly weaker than the control. Ababutain found that the strength of inhibition of basil leaf extracts against *S. aureus*, *Bacillus subtilis*, *E. coli* and *P. aeruginosa* was in the order methanol > ethanol > water extract, and that all extracts had the same effect on the yeasts *Candida albicans* and *C. tropicalis* [106]. In his study, even the erythromycin-resistant strains of *S. aureus* and *E. coli* were sensitive to all *O. basilicum* extracts.

Fungi play various roles in food, from production to spoilage. Every year, millions of people fall ill from foodborne diseases, some of which are caused by fungi such as *Alternaria*, *Aspergillus*, *Candida* and *Fusarium,* which mainly affect immunocompromised individuals [107]. The importance of plant extracts as a novel approach to combat pathogenic microorganisms is increasingly being recognised. In this context, polyphenols, which are found in all higher plants, have been extensively studied for their antimicrobial properties. It is assumed that their mode of action is based on their ability to directly combat microorganisms and suppress microbial virulence factors [108]. In this study, the extracts contained a variety of phenolic compounds, such as flavonoids and a phenolic diterpene (carnosic acid), while phenolic acids were only present in the ethanol extract. Bais et al. reported an increased production of rosmarinic acid in hairy root cultures of *O. basilicum* upon elicitation with fungal cell wall elicitors of the plant pathogen *Phytophthora cinnamomi* [109]. They also found that rosmarinic acid exerted antimicrobial activity against a range of soil-borne microorganisms, with the most detrimental effects against *P. aeruginosa* and significant inhibitory activity against *A. niger*. In addition, the methanolic-aqueous extract of *O. basilicum* was reported to exert 95.8% mycelial inhibition against the toxigenic strain of *Aspergillus flavus* and pronounced antiaflatoxigenic activity [110]. These results are in agreement with those of our study. All extracts were able to inhibit fungal strains due to the variety of specialised metabolites reported here, e.g., phenolic acids [111], methoxylated flavonoids [112], and triterpenoid acids [113]. The ethanol extract was the only one containing phenolic acids and their derivatives, of which rosmarinic acid is the most abundant in sweet basil according to literature data [14,85,88] and showed the strongest activity against *P. aeruginosa*. The acetonitrile oil extract, which exhibited strong antibacterial activity against the Gram-positive bacterium *S. aureus* and the Gram-negative bacterium *P. hauseri*, was characterised by the content of diterpenoids, especially abietane-type diterpenoids. Abietane diterpenoids from the Lamiaceae family have been reported to exhibit a range of antimicrobial activities, including antibacterial, antifungal, and antiparasitic activities [113,114].

## 5. Conclusions

In this study, solvents of different polarity (ethanol, dichloromethane and sunflower oil) were used to investigate the influence of different polarity conditions, as they may occur in food matrices during different food preparation processes, on the phytochemical composition and bioactivity of basil. The ethanol extract showed the highest antioxidant potential among the extracts. All extracts displayed notable antifungal activities against selected yeast and fungi species. More polar conditions, as found in cooking broths, lead to the extraction of (poly)phenols with significant antioxidant potential, which are important for fighting inflammation in the body. Less polar extraction methods, which can be considered analogous to stir-frying, braising/baking with vegetable oil, or marinating in oil, contribute to the extraction of nonpolar bioactive compounds, such as diterpenoids and triterpenoids.

## Figures and Tables

**Table 1:** Composition of *O. basilicum* ethanol (EE), dichloromethane (DCME), and acetonitrile oil extract (ACNE) analysed by HPLC–DAD/ESI-ToF-MS.

No	Compound Class/Name	CAS Registry Number	Molecular Formula	Ion Species	Pseudomolecular Ion (*m*/*z*)	Rt (min)		UV Maximum (nm)	Extract	Reference
						ESI^–^	LC-DAD			
	***Organic acid***									
1	Succinic acid	110-15-6	C_4_H_6_O_4_	[M−H]^−^	117.0205	2.70	2.62	192, 218, 234	EE	[27]
2	Benzoic acid	65-85-0	C_7_H_6_O_2_	[M−H]^−^	121.0297	7.78	7.66	232, 284	EE, DCME	[28]
3	12-Hydroxyjasmonic acid	140631-27-2	C_12_H_18_O_4_	[M−H]^−^ [M+HCO_2_]^−^ [2M−H]^−^	225.1141 271.1216 451.2346	8.00	n.a. ^1^	n.a.	EE, DCME	[29]
4	Cinnamic acid	621-82-9	C_9_H_8_O_2_	[M−H]^−^	147.0454	11.58	n.a.	n.a.	DCME	[17]
5	Azelaic acid	123-99-9	C_9_H_16_O_4_	[M−H]^−^	187.0982	13.50	n.a.	n.a.	EE, DCME	[30]
	***Phenolic acid (derivative)***									
6	Protocatechuic acid	99-50-3	C_7_H_6_O_4_	[M−H]^−^ [2M−H]^−^	153.0195 307.0498	4.57	4.49	232, 258, 296	EE	[31]
7	4-Hydroxybenzoic acid	99-96-7	C_7_H_6_O_3_	[M−H]^−^ [2M−H]^−^	137.0245 275.0607	5.80	5.70	238, 280, 310	EE	[31]
8	Caffeic acid	331-39-5	C_9_H_8_O_4_	[M−H]^−^	179.0354	7.16	7.06	244, 296sh, 324	EE	[32]
9	*p*-Coumaric acid	7400-08-0	C_9_H_8_O_3_	[M−H]^−^	163.0372	9.41	9.28	296sh, 310	EE	[14]
10	Rosmarinic acid	20283-92-5	C_18_H_16_O_8_	[M−H]^−^ [M+HCO_2_]^−^ [2M−H]^−^	359.0783 405.0845 719.1621	15.53	15.35	234, 288, 330	EE	[33]
11	Salvianolic acid A	96574-01-5	C_26_H_22_O_10_	[M−H]^−^	493.1158	15.88	n.a.	n.a.	EE	[34]
12	Salvianolic acid B Salvianolic acid E Salvianolic acid L	121521-90-2 142998-46-7 389065-74-1	C_36_H_30_O_16_	[M+CF_3_CO_2_]^−^	717.1473	18.91	n.a.	n.a.	EE	[14] [35] [36]
	***Flavonoid***									
	**flavanone**									
13	Eriocitrin	13463-28-0	C_27_H_32_O_15_	[M−H]^−^ [M+HCO_2_]^−^ [2M−H]^−^	595.1514 641.1612 1191.3053	9.20	n.a.	n.a.	EE	[37]
	**flavone**									
14	Vicenin 2	23666-13-9	C_27_H_30_O_15_	[M−H]^−^ [M+HCO_2_]^−^	593.1525 639.1586	13.16	n.a.	n.a.	EE	[38]
15	Apigenin	520-36-5	C_15_H_10_O_5_	[M−H]^−^	269.0466	25.64	25.30	232, 266, 336	EE	[33]
16	Cirsimaritin Pectolinarigenin Ladanein	6601-62-3 520-12-7 10176-71-3	C_17_H_14_O_6_	[M−H]^−^	313.0729	28.12	27.56	230, 258, 268, 280	EE, DCME, ACNE	[39] [40] [39]
17	Cirsilineol Eupatorin Xanthomicrol Nevadensin 5,8-Dihydroxy-4′,6,7-trimethoxy-flavone (7CI)	41365-32-6 855-96-9 16545-23-6 10176-66-6 2798-22-3	C_18_H_16_O_7_	[M−H]^−^	343.0841	29.60	n.a.	n.a.	DCME	[41] [39] [42] [39] [41]
18	Acacetin Biochanin Genkwanin Negletein	480-44-4 491-80-5 437-64-9 29550-13-8	C_16_H_12_O_5_	[M−H]^−^	283.0623	29.76	29.26	230, 282, 330	EE, DCME	[39] [43] [39] [44]
19	Cirsilineol Eupatorin Xanthomicrol Nevadensin 5,8-Dihydroxy-4′,6,7-trimethoxy-flavone (7CI)	41365-32-6 855-96-9 16545-23-6 10176-66-6 2798-22-3	C_18_H_16_O_7_	[M−H]^−^	343.0824	29.91	n.a.	n.a.	ACNE	[41] [39] [42] [39] [41]
	**flavonol**									
20	Rutin	153-18-4	C_27_H_30_O_16_	[M−H]^−^ [M+HCO_2_]^−^ [2M−H]^−^	609.1468 655.1522 1219. 2982	10.32	10.20	354, 266sh, 298sh, 352	EE	[33]
21	Kaempferol 3-*O*-rutinoside	17650-84-9	C_27_H_30_O_15_	[M−H]^−^ [2M−H]^−^	593.1525 1187.3085	10.36	n.a.	n.a.	EE	[33]
22	Quercetin 3-glucoside Hyperoside Quercetin 7-*O*-glucoside Quercetin 3-*O*-hexoside Isoquercitrin	482-35-9 482-36-0 491-50-9 21637-25-2 905846-12-0	C_21_H_20_O_12_	[M−H]^−^ [M+HCO_2_]^−^ [2M−H]^−^	463.0895 509.0958 927.1835	11.27	11.11	256, 264sh, 296sh, 352	EE	[45] [45] [43] [46] [47]
	**flavone/flavonol**									
23	Astragalin Quercitrin Luteolin 7-*O*-glucoside Luteolin 4′-*O*-glucoside Galuteolin Orientin	480-10-4 522-12-3 5373-11-5 6920-38-3 20344-46-1 28608-75-5	C_21_H_20_O_11_	[M−H]^−^ [2M−H]^−^	447.0948 895.1926	14.50	14.31	232, 266, 288, 342	EE	[43] [48] [20] [49] [50] [20]
	***Terpenoid***									
	**monoterpenoid**									
24	2-[2-(*β*-D-Glucopyra- nosyloxy)-1-meth- ylethyl]-5-methyl-cy- clohexanone [2*R*- [2*α*(R*),5*β*]]-(9CI) (3*S*,6*S*)-6-Ethenyltetra- hydro-2,2,6-trimethyl- 2*H*-pyran-3-yl *β*-D-glu- copyranoside (ACI) *p*-Menth-1-ene-3,4-diol 4-*O*-*β*-glucopyranoside Betulalbuside A (1*S*,4*R*,5*S*)-1,3,3-Trime- thyl-2-oxabicyclo [2.2.2]oct-5-yl *β*-D-glu- copyranoside (ACI) (1*R*,2*S*,4*R*,5*S*)-5-Hy- droxy-1,3,3-trime- thylbicyclo [2.2.1]hept- 2-yl *β*-D-glucopyra- noside (ACI) (1*R*,4*S*,5*S*)-1,3,3-Trime- thyl-2-oxabicyclo [2.2.2]oct-5-yl *β*-D-glu- copyranoside (ACI) (1*S*,2*S*,4*R*)-2-Hydroxy- 1,8-cineole *β*-D-gluco- pyranoside	78916-66-2 174760-79-3 403613-11-6 64776-96-1 2104786-86-7 217960-83-3 155836-27-4 113270-15-8	C_16_H_28_O_7_	[M+HCO_2_]^−^	377.1829	8.32	n.a.	n.a.	EE	[51] [52] [53] [54] [55] [56] [57] [58]
25	2-[2-(*β*-D-Glucopyra- nosyloxy)-1-meth- ylethyl]-5-methyl-cy- clohexanone [2*R*- [2*α*(R*),5*β*]]-(9CI) (3*S*,6*S*)-6-Ethenyltetra- hydro-2,2,6-trimethyl- 2*H*-pyran-3-yl *β*-D-glu- copyranoside (ACI) *p*-Menth-1-ene-3,4-diol 4-*O*-*β*-glucopyranoside Betulalbuside A (1*S*,4*R*,5*S*)-1,3,3-Trime- thyl-2-oxabicyclo [2.2.2]oct-5-yl *β*-D-glu- copyranoside (ACI) (1*R*,2*S*,4*R*,5*S*)-5-Hy- droxy-1,3,3-trime- thylbicyclo [2.2.1]hept- 2-yl *β*-D-glucopyra- noside (ACI) (1*R*,4*S*,5*S*)-1,3,3-Trime- thyl-2-oxabicyclo [2.2.2]oct-5-yl *β*-D-glu- copyranoside (ACI) (1*S*,2*S*,4*R*)-2-Hydroxy- 1,8-cineole *β*-D-gluco- pyranoside	78916-66-2 174760-79-3 403613-11-6 64776-96-1 2104786-86-7 217960-83-3 155836-27-4 113270-15-8	C_16_H_28_O_7_	[M+HCO_2_]^−^	377.1826	9.17	n.a.	n.a.	EE	[51] [52] [53] [54] [55] [56] [57] [58]
26	(-)-*α*-Terpineol 8-*O*-*β*-D-glucopyranoside Geranyl glucoside Neryl glucoside Linalool glucoside	89616-07-9 22850-13-1 22850-14-2 82928-12-9	C_16_H_28_O_6_	[M+HCO_2_]^−^	361.1878	22.70	n.a.	n.a.	EE, DCME	[59] [60] [61] [58]
	**sesquiterpenoid**									
27	Roseoside	54835-70-0	C_19_H_30_O_8_	[M+HCO_2_]^−^	431.1936	6.69	n.a.	n.a.	EE	[45]
	**diterpenoid**									
28	Carnosic acid	3650-09-7	C_20_H_28_O_4_	[M−H]^−^	331.1919	29.65	n.a.	n.a.	ACNE	[20]
29	15-*Nor*-14-oxolabda-8(17),12*E*-diene-18-oic acid *ent*-15-*Nor*-14-oxolabda-8(17),12*E*-dien-18-oic acid	1039673-32-9 81920-05-0	C_19_H_28_O_3_	[M−H]^−^	303.1957	30.55	n.a.	n.a.	ACNE	[62] [63]
30	Royleanone	6812-87-9	C_20_H_28_O_3_	[M−H]^−^	315.1975	32.25	n.a.	n.a.	ACNE	[64]
31	Bodinieric acid A	2227130-30-3	C_19_H_24_O_4_	[M−H]^−^	315.1616	33.43	n.a.	n.a.	DCME	[65]
32	Odonicin	51419-51-3	C_24_H_30_O_7_	[M−H]^−^	429.1914	33.76	n.a.	n.a.	ACNE	[66]
33	Lagopsin C 15-*epi*-Lagopsin C Lagopsin D 15-*epi*-Lagopsin D Sideripullol C	1590387-64-6 1590387-65-7 1590387-66-8 1590387-67-9 1621480-82-7	C_22_H_36_O_6_	[M−H]^−^	395.2454	34.29	33.60	258sh, 276	DCME	[67] [67] [67] [67] [68]
34	1,4-Phenanthrenedione, 10-butoxy-4b,5,6,7,8,8a,9,10-octahydro-3-hydroxy-4b,8,8-trimethyl-2-(1-methylethyl)-, (4b*S*,8a*S*,10*R*)-(ACI)	3024059-23-9	C_24_H_36_O_4_	[M−H]^−^	387.2531	36.11	n.a.	n.a.	ACNE	[69]
35	Palustric acid	1945-53-5	C_20_H_30_O_2_	[M−H]^−^	301.2168	36.47	n.a.	n.a.	ACNE	[70]
	**sesterpenoid**									
36	Leucosceptroid B (1*R*,3*S*,3a*R*,4a*S*,5*S*,7a*S*,8*S*,8a*R*)-Decahydro-8a-hydroxy-3,5,8-trimethyl-3-[2-(3-methyl-2-furanyl)ethyl]-1-(2-methyl-1-propen-1-yl)-4*H*-cyclopent[f]isobenzofuran-4-one (ACI)	1239975-37-1 1443528-32-2	C_25_H_36_O_4_	[M+HCO_2_]^−^	399.2555	36.81	n.a.	n.a.	ACNE	[71] [72]
	**triterpenoid**									
37	Vitexnegheteroin H Sanguic acid	2173172-57-9 821797-62-0	C_30_H_46_O_7_	[M−H]^−^ [2M−H]^−^	517.3185 1035; 6401	28.68	n.a.	n.a.	EE	[73] [74]
38	Madecassic acid	18449-41-7	C_30_H_48_O_6_	[M−H]^−^ [2M−H]^−^	503.3388 1007; 6836	28.81	n.a.	n.a.	EE, DCME	[75]
39	Euscaphic acid Tormentic acid	53155-25-2 13850-16-3	C_30_H_48_O_5_	[M−H]^−^	487.3441	30.90	30.62	196, 210, 218, 228	EE, DCME	[76] [77]
40	Euscaphic acid Tormentic acid	53155-25-2 13850-16-3	C_30_H_48_O_5_	[M−H]^−^ [M+HCO_2_]^−^	487.3426 533.3427	30.98	n.a.	n.a.	ACNE	[76] [77]
41	Alphitolic acid Pomolic acid 3-*epi*-Maslinic acid	19533-92-7 13849-91-7 26563-68-8	C_30_H_48_O_4_	[M−H]^−^	471.3491	31.02	n.a.	n.a.	DCME	[76,77] [77] [76]
42	Glycyrrhetinic acid	471-53-4	C_30_H_46_O_4_	[M−H]^−^	469.3339	33.98	n.a.	n.a.	DCME	[78]
43	Alphitolic acid Pomolic acid 3-*epi*-Maslinic acid	19533-92-7 13849-91-7 26563-68-8	C_30_H_48_O_4_	[M−H]^−^	471.3493	34.48	33.74	206, 212, 216sh, 280	EE, DCME	[76,77] [77] [76]
44	Alphitolic acid Pomolic acid 3-*epi*-Maslinic acid	19533-92-7 13849-91-7 26563-68-8	C_30_H_48_O_4_	[M−H]^−^	471.3465	34.70	n.a.	n.a.	ACNE	[76,77] [77] [76]
45	Oleanolic acid (+)-Ursolic acid 3-*epi*-Ursolic acid Betulinic acid	508-02-1 77-52-1 989-30-0 472-15-1	C_30_H_48_O_3_	[M−H]^−^	455.3546	37.45	n.a.	n.a.	DCME	[79] [20,33] [80] [20]
	***Fatty acid***									
46	FA ^2^ 18:2;O3		C_18_H_32_O_5_	[M−H]^−^ [M+HCO_2_]^−^	327.2188 373.2204	25.33	24.98	234, 292	EE, DCME	
47	FA 18:2;O3		C_18_H_32_O_5_	[M−H]^−^	327.2187	25.64	n.a.	n.a.	EE, DCME	
48	FA 18:2;O3		C_18_H_32_O_5_	[M−H]^−^	327.2181	25.84	n.a.	n.a.	EE, DCME	
49	FA 18:2;O3		C_18_H_32_O_5_	[M−H]^−^	327.2191	26.20	25.60	250	EE, DCME	
50	FA 18:1;O3		C_18_H_34_O_5_	[M−H]^−^	329.2345	26.52	26.31	234, 282	EE, DCME	
51	FA 18:2;O3		C_18_H_32_O_5_	[M−H]^−^	327.2192	26.90	n.a.	n.a.	DCME	
52	FA 16:0;O2		C_16_H_32_O_4_	[M−H]^−^	287.2241	27.40	27.26	232, 258, 268, 278	DCME	
53	FA 18:3;O2		C_18_H_30_O_4_	[M−H]^−^	309.2080 355.2159	27.88	27.57	260, 268, 278	DCME	
54	FA 18:3;O2		C_18_H_30_O_4_	[M−H]^−^	309.2080 355.2159	28.06	27.74	258, 268, 278	DCME	
55	FA 18:3;O3		C_18_H_30_O_5_	[M−H]^−^	325.2031	28.28	n.a.	n.a.	EE, DCME	
56	FA 18:4;O2		C_18_H_28_O_4_	[M−H]^−^ [M+HCO_2_]^−^ [2M−H]^−^	307.1925 353.1996 615.3859	28.47	28.15	314	EE, DCME	
57	FA 18:4;O2		C_18_H_28_O_4_	[M−H]^−^	307.1925	28.64	28.28	254sh, 320	EE, DCME	
58	FA 18:3;O4		C_18_H_30_O_6_	[M−H]^−^	341.1982	28.87	n.a.	n.a.	EE	
59	FA 17:3;O3 FA 16:3;O		C_17_H_28_O_5_ C_16_H_26_O_3_	[M−H]^−^ [M+HCO_2_]^−^	311.1878	29.04	28.69	264, 280, 334	DCME	
60	FA 18:3;O3		C_18_H_30_O_5_	[M−H]^−^ [M+HCO_2_]^−^	325.2035 371.2107	29.22	n.a.	n.a.	DCME	
61	FA 18:3;O2		C_18_H_30_O_4_	[M−H]^−^	309.2081	29.42	n.a.	n.a.	DCME	
62	FA 18:3;O2		C_18_H_30_O_4_	[M−H]^−^ [M+HCO_2_]^−^	309.2080 355.2110	29.58	n.a.	n.a.	DCME	
63	FA 18:3;O2		C_18_H_30_O_4_	[M−H]^−^ [M+HCO_2_]^−^	309.2080 355.2122	29.80	n.a.	n.a.	EE, DCME	
64	FA 18:3;O2		C_18_H_30_O_4_	[M−H]^−^	309.2069	29.80	n.a.	n.a.	ACNE	
65	FA 18:2;O2		C_18_H_32_O_4_	[M−H]^−^ [M+HCO_2_]^−^	311.2238 357.2308	29.94	29.44	262sh, 270, 282sh, 338	EE, DCME	
66	FA 18:2;O2		C18H32O4	[M−H]^−^	311.2225	30.13	n.a.	n.a.	ACNE	
67	FA 18:2;O4		C_18_H_32_O_6_	[M−H]^−^	343.2143	30.31	n.a.	n.a.	DCME	
68	FA 18:3;O2		C_18_H_30_O_4_	[M−H]^−^	309.1802	30.62	n.a.	n.a.	DCME	
69	FA 18:3;O3		C_18_H_30_O_5_	[M−H]^−^	325.2017	30.66	n.a.	n.a.	ACNE	
70	FA 18:1;O2		C_18_H_34_O_4_	[M−H]^−^	313.2379	30.90	n.a.	n.a.	ACNE	
71	FA 18:1;O2		C_18_H_34_O_4_	[M−H]^−^	313.2396	31.15	n.a.	n.a.	EE, DCME	
72	FA 18:1;O2		C_18_H_34_O_4_	[M−H]- [M+HCO_2_]^−^	313.2373 359.2435	31.37	30.91	282	ACNE	
73	FA 18:1;O2		C_18_H_34_O_4_	[M−H]^−^	313.2381	32.15	n.a.	n.a.	ACNE	
74	FA 18:3;O		C_18_H_30_O_3_	[M−H]^−^ [M+HCO_2_]^−^	293.2124 339.2205	32.18	31.56	276, 332, 410	EE, DCME	
75	FA 18:3;O		C_18_H_30_O_3_	[M−H]^−^	293.2112	32.31	31.71	230, 278, 286sh	DCME	
76	FA 18:3;O		C_18_H_30_O_3_	[M−H]^−^	293.2113	32.46	n.a.	n.a.	ACNE	
77	FA 18:3;O2		C_18_H_30_O_4_	[M−H]^−^ [M+HCO_2_]^−^ [2M−H]^−^	309.2082 355.2160 619.4192	32.55	31.90	200, 218sh, 232	EE, DCME	
78	FA 18:0;O2		C_18_H_36_O_4_	[M−H]^−^ [M+HCO_2_]^−^	315.2528 361.2556	32.64	n.a.	n.a.	DCME, ACNE	
79	FA 18:1;O3		C_18_H_34_O_5_	[M−H]^−^	329.1906	33.05	32.45	202, 212, 220	EE	
80	FA 18:3;O		C_18_H_30_O_3_	[M−H]^−^	293.2112	33.06	n.a.	n.a.	DCME	
81	FA 18:2;O		C_18_H_32_O_3_	[M−H]^−^ [M+HCO_2_]^−^	295.2294 341.2336	33.36	32.63	280, 414	EE, DCME	
82	FA 18:1;O3		C_18_H_34_O_5_	[M−H]^−^	329.1889	33.36	n.a.	n.a.	ACNE	
83	FA 18:2;O2		C_18_H_32_O_4_	[M−H]^−^	311.2241	33.52	n.a.	n.a.	EE	
84	FA 18:3;O		C_18_H_30_O_3_	[M−H]^−^	293.2133	33.55	n.a.	n.a.	EE, DCME	
85	FA 18:2;O2		C_18_H_32_O_4_	[M−H]^−^	311.2241	33.61	n.a.	n.a.	DCME	
86	FA 18:2;O		C_18_H_32_O_3_	[M−H]^−^	295.2271	33.65	n.a.	n.a.	ACNE	
87	FA 18:2;O		C_18_H_32_O_4_	[M−H]^−^	311.2242	33.66	33.04	208, 214, 220sh	EE	
88	FA 18:3;O		C_18_H_30_O_3_	[M−H]^−^	293.2135	33.83	33.05	234, 268, 326	DCME	
89	FA 18:2;O2		C_18_H_32_O_4_	[M−H]^−^	311.2227	33.90	n.a.	n.a.	ACNE	
90	FA 18:3;O		C_18_H_30_O_3_	[M−H]^−^	293.2133	34.10	33.43	204, 216, 280	EE, DCME, ACNE	
91	FA 18:3;O		C_18_H_30_O_3_	[M−H]- [M+HCO_2_]^−^	293.2116 339.2167	34.34	n.a.	n.a.	ACNE	
92	FA 18:1;O		C_18_H_34_O_3_	[M−H]^−^	297.2429	34.37	n.a.	n.a.	ACNE	
93	FA 18:1;O		C_18_H_34_O_3_	[M−H]^−^	297.2449	34.55	n.a.	n.a.	DCME	
94	FA 18:1;O2		C_18_H_34_O_4_	[M−H]^−^ [M+HCO_2_]^−^	313.2402 359.2452	34.65	n.a.	n.a.	EE, DCME	
95	FA 18:3;O		C_18_H_30_O_3_	[M−H]^−^ [M+HCO_2_]^−^	293.2115 339.2178	34.75	34.36	276	EE, DCME, ACNE	
96	FA 18:1;O2		C_18_H_34_O_4_	[M−H]^−^	313.2401	34.82	34.04	276	EE, DCME	
97	FA 18:2;O		C_18_H_32_O_3_	[M−H]^−^	295.2271	34.91	n.a.	n.a.	ACNE	
98	FA 18:2;O		C_18_H_32_O_3_	[M−H]^−^	295.2268	35.15	n.a.	n.a.	ACNE	
99	FA 18:1;O		C_18_H_34_O_3_	[M−H]^−^	297.2429	36.32	n.a.	n.a.	ACNE	
100	FA 18:3		C_18_H_30_O_2_	[M−H]^−^ [M+HCO_2_]^−^	277.2194 323.2194	36.47	n.a.	n.a.	EE, DCME	
101	FA 18:3		C_18_H_30_O_2_	[M−H]^−^ [M+HCO_2_]^−^	277.2166 323.2261	36.80	n.a.	n.a.	ACNE	
102	FA 18:2		C_18_H_32_O_2_	[M−H]^−^ [M+HCO_2_]^−^	279.2323 325.2383	37.83	n.a.	n.a.	ACNE	
103	Undecanedioic acid	1852-04-6	C_11_H_20_O_4_	[M−H]^−^	215.1295	24.94	24.62	202, 208, 214, 232	EE, DCME	[81]
	***Glycosylmonoacylglycerol***									
104	Gingerglycolipid A	145937-22-0	C_33_H_56_O_14_	[M−H]^−^ [M+HCO_2_]^−^ [2M−H]^−^	675.3613 721.3657 1351.7256	31.32	n.a.	n.a.	EE	[82]
105	Panaxcerol B	171520-42-6	C_27_H_46_O_9_	[M−H]^−^	513.3079 559.3137	33.07	n.a.	n.a.	EE, DCME	[82]
	***Monoacylglycerol***									
106	2-Monolinolein	3443-82-1	C_21_H_38_O_4_	[M−H]^−^	353.2718	37.16	n.a.	n.a.	ACNE	[83]
	***Coumarin***									
107	Esculetin	305-01-1	C_9_H_6_O_4_	[M−H]^−^	177.0197	6.92	n.a.	n.a.	EE	[38]
	***Xanthone***									
108	Moreollic acid	173792-68-2	C_34_H_40_O_9_	[M−H]^−^ [2M−H]^−^	591.2630 1183.5283	35.62	n.a.	n.a.	DCME	[83]
	***Neolignan***									
109	Dehydrodieugenol B	75225-33-1	C_20_H_22_O_4_	[M−H]^−^	325.1442	32.89	n.a.	n.a.	ACNE	[84]

^1^ n.a.—Not available; ^2^ FA—Fatty acid.

**Table 2:** DPPH antiradical activity (EC_50_, mg/mL) of *O. basilicum* ethanol (EE), dichloromethane (DCME), acetonitrile oil (ACNE), and oil extract (OE); BHT—butylated hydroxytoluene.

	EE	DCME	ACNE	OE	BHT (Methanol)	BHT (Toluene)
EC_50_^1^ (mg/mL)	1.73 ± 0.06	9.55 ± 0.15	15.92 ± 0.31	52.69 ± 1.39	0.33 ± 0.01	1.42 ± 0.01

^1^ Data are presented as the mean ± standard deviation (*n* = 3).

**Table 3:** Antioxidant capacity of the extracts expressed in µg BHTE/mg extract; different letters indicate statistically significant differences (*p* ≤ 0.05).

	EE	DCME	ACNE	OE
BHTE ^1^	189.27 ± 6.61 ^a^	149.19 ± 1.71 ^b^	89.49 ± 1.73 ^c^	27.05 ± 0.72 ^d^

^1^ Data are presented as mean ± standard deviation (*n* = 3).

**Table 4:** Antibacterial activity of *O. basilicum* ethanol (EE), dichloromethane (DCME), acetonitrile oil (ACNE), and oil extract (OE) against Gram-negative bacteria. MIC—minimum inhibitory concentration; MBC—minimum bactericidal concentration.

	*Escherichia coli*		*Pseudomonas aeruginosa*		*Proteus hauseri*		*Klebsiella pneumoniae*		*Salmonella enterica* subsp. *enterica*	
	MIC	MBC	MIC	MBC	MIC	MBC	MIC	MBC	MIC	MBC
	mg/mL		mg/mL		mg/mL		mg/mL		mg/mL	
EE	2.5	>10	0.313	1.25	2.5	>10	0.625	2.5	0.625	2.5
DCME	/	/	/	/	/	/	/	/	/	/
ACNE	2.5	>10	1.25	5	0.313	1.25	0.625	1.25	1.25	5
OE	1.25	10	1.25	5	2.5	10	2.5	10	0.625	2.5
Chloramphenicol	0.062		0.25		0.125		0.062		0.125	

**Table 5:** Antibacterial activity of *O. basilicum* ethanol (EE), dichloromethane (DCME), acetonitrile oil (ACNE), and oil extract (OE) against Gram-positive bacteria. MIC—minimum inhibitory concentration; MBC—minimum bactericidal concentration.

	*Staphylococcus aureus*		*Bacillus subtilis*		*Clostridium sporogenes*	
	MIC	MBC	MIC	MBC	MIC	MBC
	mg/mL		mg/mL		mg/mL	
EE	0.625	2.5	1.25	10	0.625	2.5
DCME	/	/	/	/	/	/
ACNE	0.313	1.25	1.25	5	2.5	10
OE	2.5	10	2.5	10	2.5	10
Chloramphenicol	0.015		0.015		0.25	

**Table 6:** Antifungal activity of *O. basilicum* ethanol (EE), dichloromethane (DCME), acetonitrile oil (ACNE), and oil extract (OE). MIC—minimum inhibitory concentration; MFC—minimum fungicidal concentration.

	*Aspergillus brasiliensis*		*Saccharomyces cerevisiae*		*Candida albicans*	
	MIC	MFC	MIC	MFC	MIC	MFC
	mg/mL		mg/mL		mg/mL	
EE	1.25	>10	1.25	10	2.5	5
DCME	1.25	5	1.25	5	2.5	5
ACNE	1.25	5	1.25	5	2.5	5
OE	1.25	10	1.25	5	2.5	5
Nystatin	2.5		1.25		2.5	

## Data Availability

The original contributions presented in the study are included in the article, further inquiries can be directed to the corresponding author.
